# Practical considerations for laboratories: Implementing a holistic quality management system

**DOI:** 10.3389/fbioe.2022.1040103

**Published:** 2022-11-03

**Authors:** Segaran Pillai, Jennifer Calvert, Elizabeth Fox

**Affiliations:** ^1^ Office of Laboratory Safety, Office of The Commissioner, Food and Drug Administration, Washington D.C., MD, United States; ^2^ Booz Allen Hamilton, McLean, Tysons Corner, VA, United States

**Keywords:** quality, LQMS, QMS, laboratory, implementation, framework, total quality, reproducibility

## Abstract

A laboratory quality management system (LQMS) is an essential element for the effective operation of research, clinical, testing, or production/manufacturing laboratories. As technology continues to rapidly advance and new challenges arise, laboratories worldwide have responded with innovation and process changes to meet the continued demand. It is critical for laboratories to maintain a robust LQMS that accommodates laboratory activities (e.g., basic and applied research; regulatory, clinical, or proficiency testing), records management, and a path for continuous improvement to ensure that results and data are reliable, accurate, timely, and reproducible. A robust, suitable LQMS provides a framework to address gaps and risks throughout the laboratory path of workflow that could potentially lead to a critical error, thus compromising the integrity and credibility of the institution. While there are many LQMS frameworks (e.g., a model such as a consensus standard, guideline, or regulation) that may apply, ensuring that the appropriate framework is adopted based on the type of work performed and that key implementation steps are taken is important for the long-term success of the LQMS and for the advancement of science. Ultimately, it ensures accurate results, efficient operations, and increased credibility, enabling protection of public health and safety. Herein, we explore LQMS framework options for each identified laboratory category and discuss prerequisite considerations for implementation. An analysis of frameworks’ principles and conformity requirements demonstrates the extent to which they address basic components of effective laboratory operations and guides optimal implementation to yield a holistic, sustainable framework that addresses the laboratory’s needs and the type of work being performed.

## Introduction

In order for laboratories to support the public health mission and better address emerging public health challenges, the need for an LQMS that facilitates risk-based thinking and enhances assurance of data quality becomes more critical. With the worldwide shock of the coronavirus disease 2019 (COVID-19) outbreak, organizations have been forced to pivot and innovate to address new issues. The rapid response across industries towards COVID-19 and other crises illustrates business resilience and emphasizes the importance of quality management in the laboratory and its organizational culture. However, incidents stemming from gaps in quality management and affecting public health continue to occur. Laboratory errors have a reported frequency of 0.012%–0.6% of all test results, which in turn has huge impact on diagnosis, as 60%–70% of all diagnoses are made based upon laboratory tests (Agarwal, 2014). In addition, there is evidence of an observed data reproducibility crisis in the research community that would benefit from attention and improvement. A recent *Nature* survey of 1,576 researchers found that 52% of those surveyed agree that there is a crisis of reproducibility; the same survey found that over 70% have failed to reproduce another scientist’s data (Baker, 2016). There are numerous benefits of an LQMS that contribute to managing risks in the laboratory, including errors, and mitigating reproducibility crisis concerns. One major benefit is enabling the laboratory to better identify, assess, and address risks faced in the laboratory at all levels of the organization. Poor data quality is one such risk. Some standards, such as International Organization for Standardization (ISO) 31,000, are completely dedicated to risk management (International Organization for Standardization, 2018), while other more holistic quality management standards, guidelines, and regulations, such as ISO 17025, incorporate the concept of risk-based thinking throughout their respective frameworks (International Organization for Standardization, 2017). In turn, risk management and other aspects of quality management can be built into all three phases of the workflow path (commonly referred to as the Total Testing Process) of a laboratory: pre-analytical, analytical, and post-analytical (World Health Organization, 2011):

• The Pre-analytic phase of a laboratory’s workflow encompasses the activities that are completed prior to operational testing and research (e.g., sample collection and transport). These activities are performed to prepare and support the laboratory in its operations, research, and services.
• The Analytic phase of a laboratory’s workflow encompasses the activities that are completed within the laboratory during operational testing and research. These activities are performed to directly execute the laboratory’s operations, research, and services (e.g., sample testing, experimental studies).
• The Post-analytic phase of a laboratory’s workflow encompasses the activities that are completed after operational testing and research (e.g., reporting). These activities assure that processes are conducted in a manner that ensures compliance, accuracy, customer satisfaction, and continual improvement.

Each phase must be addressed when implementing an LQMS. While one may deduce that the highest risk of error would occur in the analytic phase, there is now incontrovertible evidence that the majority of laboratory errors occur in the pre-analytical phase (61.9%–68.2%), which are followed by mistakes in the post-analytical phase (18.5%–23.1%) and analytical phase (13.3%–15%) (Mrazek et al., 2020). Within the path of workflow, laboratory processes and procedures can be organized into 12 management areas, known as Quality System Essentials (QSEs); the 12 QSEs are globally recognized principles that address all aspects of an LQMS and cover the entire path of workflow of a laboratory. When all of the laboratory procedures and processes are organized into an understandable and workable structure, the opportunity to ensure that all are appropriately managed is increased (World Health Organization, 2011). Moreover, as safety is a key QSE (QSE 2: Facilities and Safety), laboratories such as those handling pathogens and other biosafety risks have the opportunity to evaluate the effectiveness of their biosafety procedures and implement improvements (e.g., take measures to reduce the risk of contamination). An integrated, robust LQMS can help the laboratory cope with uncertainties that naturally occur in all laboratory environments. In his 1993 book ‘‘Preventing Chaos in a Crisis: Strategies for Prevention, Control, and Damage Limitation,’’ Patrick Lagadec emphasizes that the response to an emergency cannot be developed unless the institution has prepared to adapt:

**‘‘**the ability to deal with a crisis situation is largely dependent on the structures that have been developed before chaos arrives. The event can in some ways be considered as an abrupt and brutal audit: at a moment’s notice, everything that was left unprepared becomes a complex problem, and every weakness comes rushing to the forefront” (Lagadec, 1993).

Given the naturally complex operations of the modern laboratory, minimizing preventable problems and applying lessons learned should be a primary goal.

## Defining major laboratory categories

Laboratories are distributed widely across the United States. and deal with diverse and unique challenges as a result of differing locations, type of work being performed, and federal, state and local regulations. The role of modern laboratories is quickly evolving as the delivery of health care undergoes drastic changes in the face of unprecedented challenges. With drastic changes comes the need to effectively manage them to ensure continued quality of products and services. It will be critical for a laboratory, belonging to any of the below categories, to reevaluate its alignment with the organization’s strategic direction and quality policy, continually assess risk and impact of changes, carefully define organizational roles and responsibilities, and foster a strong culture of quality and safety.

### Testing laboratories (including regulatory laboratories)

Testing laboratories conduct conformance testing to ensure the safety, efficacy, and security of a vast array of materials, including, but not limited to, human and veterinary drugs, biological products, medical devices, food, water, cosmetics, radiation-emitting products, and tobacco products. Testing laboratories are driven by and generate results to support statutory obligations to protect the public and minimize risk associated with such products or materials.

### Product development and manufacturing laboratories

Product development and manufacturing laboratories are a subset of testing laboratories that conduct routine quality control analysis, as well as pre-market research and development analysis during design phases, in order to ensure conformity of manufactured products (e.g., test reagents, devices to support analysis, testing or diagnostics) against a set of criteria or attributes selected and agreed upon by the institution. Product development and manufacturing laboratories are driven largely by regulatory requirements or customer requirements and satisfaction.

### Basic and applied research laboratories

Research laboratories can be divided into basic and applied research units. While there is some natural overlap between the two, there are key differences. Basic research is conducted to seek understanding and form answers to scientific questions. According to the Association of American Medical Colleges (AAMC), basic science research—often called fundamental or bench research—provides the foundation of knowledge for the applied science that follows^1^. Applied research laboratories conduct studies that are designed to solve practical problems. While considered separate entities from regulatory laboratories, basic and applied research laboratories conduct their work to support and inform the understanding of science, (such as pathogenesis, side effects, efficacy of medical countermeasures, development and evaluation of technology and devices, etc.) and to support regulatory requirements.

### Proficiency testing laboratories

Proficiency testing laboratories assess the performance of individual laboratories responsible for specific tests or measurements generating data for regulatory consideration. Results are used to evaluate laboratories’ continuing performance, an important aspect of laboratory quality management and ensuring data integrity.

### Clinical laboratories

Clinical laboratories conduct a variety of testing on clinical specimens to evaluate patients’ health. Their work aids in the diagnosis, treatment, and prevention of disease. The Centers for Medicare & Medicaid Services (CMS) regulates clinical laboratory testing performed on human specimens in the United States. through the Clinical Laboratory Improvement Amendments (CLIA) (Center for Medicare and Medicaid Services, 2020).

## Recommendation—Identify the correct laboratory quality management system framework for the type of laboratory work conducted

Laboratories should strive to gain a thorough understanding of their current state, goals, and objectives prior to selecting and implementing an LQMS framework. Determining the major category of the laboratory will help to narrow the focus of the target state and identify if there is any overlapping of categorization that can be addressed in different ways. Capturing the current needs of the laboratory, customers/stakeholders and their requirements, and applicable rules and regulations for which compliance is required, will help the laboratory to understand the current state in order to identify the most suitable standard or framework that will govern their LQMS and serve as the benchmark for the target state. Once the laboratory requirements are understood, an appropriate framework or standard for the LQMS can then be selected. The subsequent sections provide an analysis of well-known LQMS frameworks that can serve to guide laboratories in selecting the most appropriate model for implementation, beginning with a framework crosswalk analysis.

## Crosswalk of the quality system essentials and quality management standard requirements

Table 1 presents a crosswalk analysis of well-known quality management framework documents against the 12 QSE framework. These QSEs cover all basic elements of an LQMS. Each standard was reviewed against the QSE framework, aligning a section (e.g., clause, subclause, subpart) to the applicable QSE in order to estimate the coverage of laboratory quality for the given quality management standard.

**TABLE 1:** Crosswalk of Common Quality Management Framework Documents* *Note: This table displays a non-exhaustive list of example applicable clauses, subclauses, subparts, and requirements.

QSE	GLP (21 CFR 58)	ISO 9001:2015	ISO 17025:2017	ISO 17043:2010	CLIA (42 CFR 493)
1. Organization	• Subpart B (all)	• Clause 4 (all)	• Subclause 4.1	• Subclause 4.1	• Subpart K: 493.1200-.1239; 493.1242-.1249; 493.1282; 493.1289; 493.1299
		• Clause 5 (all)	• Subclause 4.2	• Subclause 4.2	
		• Clause 6 (all)	• Clause 5 (all)	• Subclause 4.10	
		• Subclause 7.1: 7.1.1	• Subclause 8.1	• Subclause 5.1	
		• Subclause 7.4	• Subclause 8.2	• Subclause 5.2	
			• Subclause 8.5	• Subclause 5.10	
			• Subclause 8.6	• Subclause 5.15	
			• Subclause 8.9		
2. Facilities and safety	• Subpart B: § 58.29	• Subclause 7.1: 7.1.3; 7.1.4	• Subclause 6.1	• Subclause 4.3	• Subpart J: 493.1100-493.1101
	• Subpart C (all)		• Subclause 6.3		
3. Equipment	• Subpart D	• Subclause 7.1: 7.1.3; 7.1.5.1; 7.1.5.2	• Subclause 6.4	• Subclause 4.3	• Subpart K: 493.1252-.1255
			• Subclause 6.5	• Subclause 5.6: 5.6.2	
4. Purchasing and inventory	• Subpart E: § 58.83	• Subclause 8.4	• Subclause 6.1	• Subclause 5.5	• Subpart K: 493.1252
			• Subclause 6.6		
			• Subclause 7.1:7.1.1	• Subclause 5.6	
			• Subclause 7.4		
5. Process management	• Subpart B: § 58.33; § 58.35	• Clause 8	• Subclause 7.1	• Subclause 4.4	• Subpart K: 493.1232; 493.1240-.1242; 493.1250-.1251; 493.1256-.1282; 493.1290
	• Subpart E (all)	• Subclause 9.1	• Subclause 7.2	• Subclause 4.5	
	• Subpart F (all)		• Subclause 7.3	• Subclause 4.6	
	• Subpart G (all)		• Subclause 7.4	• Subclause 4.7	
			• Subclause 7.6	• Subclause 4.8	
			• Subclause 7.7	• Subclause 4.9	
			• Subclause 7.8		
6. Assessments	• Subpart B: § 58.35	• Subclause 9.2	• Subclause 7.2: 7.2.2.1	• Subclause 5.14	• Subpart H: 493.801-.807
	• Subpart K (all)		• Subclause 7.7		• Subpart K: 493.1239; 493.1249; 493.1253-.1254; 493.1289; 493.1299
			• Subclause 8.8		
7. Personnel	• Subpart B (all)	• Subclause 7.1: 7.1.2; 7.1.6	• Subclause 6.2	• Subclause 4.1	• Subpart M: 493.1351-.1495
		• Subclause 7.2		• Subclause 4.2	
8. Customer satisfaction	• No subpart dedicated to customer satisfaction	• Subclause 4.2	• Subclause 5.4	• Subclause 4.9	• Subpart K: 493.1233-.1234; 493.1291e
		• Subclause 5.1: 5.1.2	• Subclause 7.1	• Subclause 5.4	• Subpart M: 493.1407; 493.1419
		• Subclause 8.2	• Subclause 7.9	• Subclause 5.7	
		• Subclause 8.5: 8.5.5	• Subclause 8.9: 8.9.2	• Subclause 5.8	
		• Subclause 9.1, 9.1.2, 9.1.3			
9. Occurrence management	• Subpart B: § 58.29 (f); § 58.31 (g); § 58.33 (b-c); § 58.35	• Subclause 8.7	• Subclause 7.9	• Subclause 5.8	• Subpart K: 493.1233-.1234; 493.1282
		• Subclause 10.1	• Subclause 7.10	• Subclause 5.9	
		• Subclause 10.2	• Subclause 8.7	• Subclause 5.11	
				• Subclause 5.12	
10. Continual improvement	• No subpart dedicated to continual improvement	• Subclause 6.1	• Subclause 8.5	• Subclause 5.10	• Subpart K: 493.1236-.1239; 493.1249; 493.1289; 493.1299
		• Clause 10	• Subclause 8.6	• Subclause 5.12	
11. Documents and records	• Subpart J	• Subclause 7.5	• Subclause 5.3	• Subclause 4.8	• Subpart J: 493.1101e; 493.1105
			• Subclause 7.5	• Subclause 5.2: 5.2.2	• Subpart K: 493.1283
			• Subclause 7.8	• Subclause 5.3	
			• Subclause 8.1	• Subclause 5.13	
			• Subclause 8.2		
			• Subclause 8.3		
			• Subclause 8.4		
12. Information management	• Subpart G: § 58.130 (d-e)	• Subclause 7.1: 7.1.3 d)	• Subclause 4.2	• Subclause 4.8	• Subpart K: 493.1231; 493.1291
	• Subpart J: § 58.190; § 58.195		• Subclause 7.8	• Subclause 5.13	
			• Subclause 7.11	• Subclause 4.10	

## Analysis of quality management standards by laboratory category

It is critical for a laboratory to consider the type of work conducted and resources available when selecting the most suitable framework. For example, a basic research laboratory conducting studies on plant biology may not benefit from choosing to conform to ISO 17043, a standard that specifies general requirements for proficiency testing laboratories (International Organization for Standardization, 2010). There are two basic elements for consideration of a framework for suitability: 1) its relevance to the type of work performed by the laboratory; and 2) the sustainability of the framework within the laboratory. Based on analysis of the content, potential intended uses, and approaches of quality management framework documents, Table 2 provides examples of suitable LQMS framework documents that could best apply to each major laboratory category, along with benefits and limitations determined based on the crosswalk analysis in Table 1.

**TABLE 2:** Benefits and limitations of select quality management framework documents by major laboratory category.

1	WHO LQMS handbook	ISO 9001	ISO 17025	GLP (21 CFR 58)
Testing laboratories				
Benefits	• Internationally recognized	• Internationally recognized	• Internationally recognized	• Nationally recognized by regulators and inspectors
	• All 12 QSEs and path of workflow are addressed	• Broad standard that can be applied to any organization of any size	• Developed specifically for testing and calibration laboratories	• Developed specifically for nonclinical laboratories, thus applicable to testing laboratories
	• Flexible framework that can be applied to any laboratory	• Recognized standard by regulators	• Accreditable to demonstrate additional recognition of quality	
	• Ideal for laboratories not required to be accredited to ISO or follow GLP	• Certifiable to demonstrate additional recognition of quality	• Many QSEs fully or nearly fully addressed	
Potential limitations	• Not certifiable or accreditable for customer recognition	• Not developed specifically for laboratories	• Some aspects of several QSEs not wholly addressed: Personnel, Assessments	• Not certifiable or accreditable for customer recognition
		• No guidance for laboratory processes or procedures	• Safety planning and training (as part of the Facilities & Safety QSE) not wholly addressed	• Safety planning and training (as part of the Facilities & Safety QSE) not wholly addressed
		• Some QSEs not wholly addressed: Facilities & Safety, Purchasing & Inventory, Assessments, Personnel, Information Management		• Customer Satisfaction QSE is not addressed
				• Continual Improvement QSE is not addressed

Product development and manufacturing laboratories				
	WHO LQMS handbook	ISO 9001	ISO 17025	GLP (21 CFR 58)
Benefits	• Internationally recognized	• Internationally recognized	• Internationally recognized	• Nationally recognized by regulators and inspectors
	• All 12 QSEs and path of workflow are addressed	• Broad standard that can be integrated with business processes throughout the laboratory’s institution	• Developed specifically for testing laboratories, which can be applied to conduct of manufacturing laboratories	• Developed specifically for nonclinical laboratories, thus applicable to product development and manufacturing laboratories
	• Flexible framework that can be applied to any laboratory	• Recognized standard by technical customers	• Accreditable to demonstrate additional recognition of quality
	• Ideal for laboratories not required to be accredited to ISO or follow GLP	• Certifiable to demonstrate additional recognition of quality	• Many QSEs fully or nearly fully addressed	
Potential limitations	• Not certifiable or accreditable for customer recognition	• Not developed specifically for laboratories	• Some aspects of the standard (e.g., requirements for calibration certificates) may not be applicable to product development and manufacturing laboratories	• Not certifiable or accreditable for customer recognition
		• No guidance for laboratory processes or procedures	• Several QSEs not wholly addressed: Personnel, Assessments	• Safety planning and training (as part of the Facilities & Safety QSE) not wholly addressed
		• Some QSEs not wholly addressed: Facilities & Safety, Purchasing & Inventory, Assessments, Personnel, Information Management	• Safety planning and training (as part of the Facilities & Safety QSE) not wholly addressed	• Customer Satisfaction QSE is not addressed
				• Continual Improvement QSE is not addressed

Basic and applied research laboratories				
	WHO LQMS handbook	GLP (21 CFR 58)	ISO 17025	ISO 9001
Benefits	• Internationally recognized	• Nationally recognized	• Internationally recognized	• Internationally recognized
	• All 12 QSEs and path of workflow are addressed	• Developed specifically for nonclinical laboratories, thus applicable to research laboratories	• Provides a framework to assure the quality of results, as well as the quality of externally provided products that affect research outputs	• Broad standard that can be applied to any organization of any size
	• Flexible framework that can be applied to any laboratory	• Contains specific guidance on conducting studies		• Certifiable to demonstrate additional recognition of quality
	• Ideal for laboratories not required to be accredited to ISO or follow GLP (e.g., basic and applied research laboratories)	• Laboratories conducting animal research may benefit from specific prescriptive guidance on animal care and facilities		
Potential limitations	• No limitations identified	• Safety planning and training (as part of the Facilities & Safety QSE) not wholly addressed	• Some aspects of the standard (e.g., reporting to customers, testing and calibration method validation) may not be applicable to research laboratories	• Not developed specifically for laboratories
		• Equipment QSE not wholly addressed (e.g., identification, qualification, and staff training on operation)	• Several QSEs not wholly addressed: Personnel, Assessments	• No guidance for laboratory processes or procedures
		• Does not address quality indicators	• Safety planning and training (as part of the Facilities & Safety QSE) not wholly addressed	• Conformity is defined by client requirements, but in research, results may not always match expectations and can be unknown
		• Does not address procedures for identifying or investigating problems (e.g., root causes analysis)		• Some QSEs not wholly addressed: Facilities & Safety, Purchasing & Inventory, Assessments, Personnel, Information Management
		• Preventive action not addressed		

Proficiency testing laboratories			
	WHO LQMS handbook	GLP (21 CFR 58)	ISO 17043
Benefits	• Internationally recognized	• Nationally recognized by regulators and inspectors	• Internationally recognized
	• All 12 QSEs and path of workflow are addressed	• Developed specifically for nonclinical laboratories, thus applicable to proficiency testing laboratories	• Developed specifically for proficiency testing laboratories
	• Flexible framework that can be applied to any laboratory		• Accreditable to demonstrate additional recognition of quality
	• Ideal for laboratories not required to be accredited to ISO or follow GLP		• Many QSEs nearly fully addressed
			• Contains specific guidance on proficiency testing schemes and data management procedures, including confidentiality of data and impartiality of the laboratory
Potential limitations	• Not certifiable or accreditable for customer recognition	• Not certifiable or accreditable for customer recognition	• Some aspects of several QSEs not wholly addressed: Equipment, Assessments, Personnel
		• Contains prescriptive requirements for animal care and conducting studies, which may not be relevant to proficiency testing laboratories	• Safety planning and training (as part of the Facilities & Safety QSE) not wholly addressed
		• Safety planning and training (as part of the Facilities & Safety QSE) not wholly addressed	
		• Customer Satisfaction QSE is not addressed	
		• Continual Improvement QSE is not addressed	

Clinical laboratories			
	WHO LQMS handbook	ISO 15189	CLIA (Certificate required)
Benefits	• Internationally recognized	• Developed specifically for medical (i.e., clinical) laboratories	• Nationally recognized regulatory requirements
	• All 12 QSEs and path of workflow are addressed	• Internationally recognized	• All 12 QSEs and path of workflow are addressed
	• Flexible framework that was based on clinical quality documents	• Accreditable to demonstrate additional recognition of quality	• Developed specifically for clinical laboratories
	• Ideal for laboratories not required to be accredited to ISO or follow GLP	• Many QSEs nearly fully addressed	• Contains many discipline-specific requirements that may be helpful guidance (e.g., histopathology)
		• Standard has a customer focus that many clinical quality documents lack	• Multiple types of certificates achievable depending on diagnostic testing performed and laboratory/personnel qualifications
Potential limitations	• Not certifiable or accreditable for customer recognition	• Facilities and Safety QSE not wholly addressed (specifically personnel safety)	• Generally, lacks customer-driven focus and more focused on process management
			• Personnel safety and retention not wholly addressed

### Key takeaways

From our analysis and crosswalk of quality documents, it was observed that only WHO’s LQMS Handbook, the ISO 15189 standard (International Organization for Standardization, 2012), and CLIA addressed the laboratory’s path of workflow (total testing process). WHO’s handbook explains this concept and its importance, which is valuable considering that error prevention in each phase helps to ensure the quality of outputs. While not written for laboratories, the ISO 9001 standard for quality management systems (International Organization for Standardization, 2015) was included in our analysis due to its broad flexibility and ability to integrate with a laboratory’s institutional processes. One notable finding in the crosswalk is that many of the international quality standards, the Food and Drug Administration (FDA) regulation document “Good Laboratory Practice for Nonclinical Laboratory Studies” (21 Code of Federal Regulations Part 58) (hereafter referred to as “GLP”) (FDA, 1978), and CLIA do not fully address personnel safety as a component of the Facilities and Safety QSE, but rather focus on facility maintenance and contamination control (this could be due to the fact that other national regulatory bodies address environmental and occupational safety, such as the Occupational Safety and Health Administration, Nuclear Regulatory Commission, and Environmental Protection Agency). Additionally, it was observed that purchasing and inventory guidance was also limited when compared to WHO’s LQMS framework. For example, while GLP includes requirements for reagent labeling, GLP does not have a requirement for inspection upon receipt or conducting routine inventory. Another key finding is that the clinical quality documents and GLP do not have as strong of a customer focus as the international quality standards; the focus in these documents is geared towards analytical or study conduct and process management. Establishing procedures on customer service or adhering to the customer service aspects of a suitable ISO standard may be advisable in any environment in order to ensure expectations are continually met. Ultimately, the findings in Tables 1, 2 demonstrate that a holistic LQMS framework complemented with guidance from additional quality documents can benefit many laboratories that aim to address all 12 QSEs.

## Recommendation—Conduct a gap analysis of the current laboratory quality management system against the selected framework

One of the first key steps needed prior to implementing a quality management system, for any organization, is conducting a gap analysis to assess the current state against the target state. Laboratories of all types can take this approach to leverage gap analysis data gathered to identify areas that need to be improved or prioritized to achieve successful implementation (and certification, if pursued). Once the major category of the laboratory is determined, needs and requirements are understood, and the correct framework is selected by the laboratory (and institution, as applicable), they can begin their gap analysis. The gap analysis involves evaluating their LQMS against the selected framework, which serves as the benchmark, to identify where processes and procedures are nonexistent or need improvement. It is recommended that the organization discuss and agree upon the prioritization of gaps that need to be addressed. The next section provides guidance as to the recommended prioritization level of QSEs for each major laboratory category.

### QSE prioritization for consideration

The 12 QSEs of an LQMS, including their key sub-elements (see Supplementary Table S1) are intended to support general quality management of laboratory operations; as such, implementation can be modified or prioritized based on laboratory work performed. The QSEs can fall into two basic levels of importance depending on the type of laboratory work being performed: beneficial and critical. Beneficial QSEs, while not as prioritized as critical QSEs, are recommended for effective, optimized implementation of an LQMS (see Supplementary Table S2 for detail and visualization).

## Recommendation—addressing gaps in standard requirements will help to minimize risk

While there are many benefits to applying each individual quality standard or guideline examined in this article, there are also potential limitations depending on the laboratory category and work performed. For example, ISO 17025 has less focus on personnel safety when compared to the LQMS framework detailed in WHO’s LQMS Handbook (World Health Organization, 2011), and the GLP regulations do not address customer satisfaction. Laboratory operations are complex and often have several different sets of requirements they must meet, along with processes and outputs they must monitor at each phase of the path of workflow in the laboratory. Therefore, applying a holistic, comprehensive system of quality management standards is recommended to incorporate different types of requirements (e.g., customer-specific requirements, clinical outcome criteria) and further reduce the risk of an occurrence or inaccurate, untimely, or unreliable results. Occurrences often take the form of laboratory errors, which can have significant consequences. Regarding clinical laboratories specifically, the focus on analytical quality for many years has resulted in less process deviations and an improved error rate that surpasses most other areas in healthcare (Hawkins, 2012). However, given the multiple lines of evidence suggesting that most errors in the total path of workflow actually fall outside of the analytical phase, and that the pre- and post-analytical steps have been found to be much more vulnerable (Lippi et al., 2013), only minor attention is still paid to these areas in many laboratories. According to a recent survey among European laboratories, about a third of the laboratories that monitor pre-analytical errors perform further analysis and, even when a statistical analysis is made, approximately 25% of them remain inactive against unsatisfactory results (Mrazek et al., 2020). Implementing a holistic LQMS helps to ensure that proper evaluation and application of corrective/preventive actions are conducted, as they are critical for prevention of recurring errors.

While a laboratory need not pursue accreditation to multiple standards, as this would require additional time and resources, many laboratories can choose the most suitable quality framework [while assuring compliance with applicable regulatory requirements (e.g., CLIA)] and incorporate aspects of others, including the plethora of standards available that were not analyzed in this article, to address gaps such as those identified in our key takeaways in section five. For example, if a testing laboratory maintains an accreditation to ISO 17025 but would like to direct additional attention towards improving personnel safety training procedures, the laboratory may consider implementing the Facilities and Safety QSE (World Health Organization, 2011) as a guideline in addition to the ISO 17025 standard. In another instance, a high-containment research laboratory that follows GLP may consider adopting elements of ISO 35001, a biorisk management standard (International Organization for Standardization, 2019). A holistic LQMS that addresses gaps can remain sustainable long-term, as it takes into consideration that risks, priorities, and needs are continuously evolving, and different standards and guidelines involved in the system can take priority in different situations; additionally, certain sources of information may be more appropriate for defining different types of criteria (Westgard, 1999). In regard to research laboratories, for which conforming entirely to a quality standard may be restrictive, a multi-faceted approach can be beneficial. A French agency that implemented a QMS for their research laboratory reported that:

‘‘although the use of a single QA [Quality Assurance] system has been proposed for both routine and research activities, it is now more and more accepted by the scientific community that specific standards are required or preferred when performing research activities. This opinion is based upon the limitations of the standards dedicated to analytical laboratories; these limitations arise from the nature of research activities and from the requirements of these standards (e.g., rigid organizational structure, pre-defined methods)” (Biré et al., 2004).

Any laboratory that has a holistic LQMS in place is prepared with methodology to adapt and address changes, new risks, and additional competencies that may be required to optimize performance.

## Discussion

There can be a persistent mindset among laboratorians that quality is about checking boxes, and if the laboratory wins certification to a standard, they’ve checked all the boxes and maintain the status quo. But quality in the laboratory is a multi-faceted system that involves much more than that to ensure continued reliability and repeatability of experiments and tests, accuracy of data, and safety of personnel. As laboratories conduct very different types of work, such as basic research, clinical testing/diagnostics, regulatory testing etc., there is currently not a one-size-fits-all approach that can be applied to all types of laboratories effectively. Digging deeper into a quality management framework and ascertaining other elements that may be needed (or conversely, aspects that would be extraneous to implement for the laboratory’s purposes) to address the complexity, nuance, and requirements of the laboratory can help to implement and sustain a successful LQMS. The findings from our analysis in Table 1 demonstrate the extent to which quality management framework documents address each of the 12 QSEs in the framework outlined in WHO’s LQMS Handbook. It was determined that not all of the QSEs are fully addressed by a single framework document (e.g., GLP does not address QSE 10: Continual Improvement), and thus a gap may exist if implemented alone. In addition, the findings in Table 2 indicate that there are different benefits and potential limitations associated with each individual quality management framework, dependent on the type of laboratory. For example, the WHO LQMS Handbook may be the most suitable framework for a basic or applied research laboratory, as limitations were not identified for this laboratory category, but this framework alone may not be the most ideal for a manufacturing laboratory that needs to be accredited to a quality standard to meet a customer requirement. From the results in both tables, we can conclude that a holistic LQMS framework complemented with guidance from multiple quality documents can benefit many laboratories that aim to address all 12 QSEs. Although a perfect LQMS is not feasible and there will always be technical and resource limitations, a holistic and robust LQMS that addresses all phases of the path of workflow to mitigate risk of errors is conceivable. A robust LQMS requires identification of the benefits, suitability and sustainability of the effort for the laboratory, a thorough understanding and incorporation of all relevant requirements, and monitoring and evaluation at the pre-analytical, analytical, and post-analytical phases. An LQMS that properly integrates all relevant requirements, promotes risk-based thinking, and addresses the entire laboratory path of workflow can ultimately promote the generation of good and relevant data, greater confidence in the quality of data and results, best practices, and a culture of responsibility and safety in the laboratory.

## Footnotes

1 Additional information can be found online at: https://www.aamc.org/what-we-do/mission-areas/medical-research/basic-science
