# Supplementary material for: Practical considerations for laboratories: Implementing a holistic quality management system
Source: Front Bioeng Biotechnol. 2022 Nov 3;10:1040103. doi: 10.3389/fbioe.2022.1040103 (PMC9670165; doi:10.3389/fbioe.2022.1040103)
Supplement: Supplementary file 1 [file DataSheet1.docx]

Supplementary Material

# Key Sub-Elements for an effective Laboratory Quality Management System (LQMS)

# Table 1 below demonstrates key areas of each of the Quality System Essentials (QSEs) that we recommend laboratories explore as part of implementation, sustainment, and continual improvement.

Table 1: Sub-Elements of the 12 QSEs

| QSE | Sub-Elements | |
| --- | --- | --- |
| 1. Organization | - Organization and structure of laboratory operations - Roles and responsibilities related to laboratory operations - Laboratory goals and objectives - Communication to ensure efficient operations - Management review of organizational structure and LQMS at planned intervals to ensure efficiency | |
| 2. Facilities & Safety | **Facilities**:   - Establishment of laboratory security procedures - Control of access to laboratory facilities - Assurance that facility design is suitable for the type of work being performed - Assurance that proper engineering controls are in place - Environmental monitoring of the laboratory and vivarium spaces (as appropriate) - Planning for continuity of operations | **Safety (establishment of procedures as applicable):**   - Biosafety procedures - Chemical hygiene procedures - Radiation safety procedures - Waste management procedures - Respiratory protection procedures - Nanomaterial safety procedures - Fire prevention procedures - Hazard identification, risk assessment, and risk mitigation procedures - Policies, manuals, plans, guides, and training - Emergency management, preparedness response, mitigation, and recovery procedures - Occupational Health Services for medical surveillance, immunization, medical evaluations, and emergency care - Proper storage and disposal of biologicals, chemicals, radiological and nanomaterials, laboratory supplies and reagents - Workplace incident and illness reporting procedures - Annual safety inspections for remedial/corrective action |
| 3. Equipment | - Qualification of equipment - Equipment inventory - Establishment of procedures for proper application and usage of laboratory equipment - Verification, validation, and/or calibration of equipment performance - Preventive maintenance of equipment - Scheduled and unscheduled maintenance of equipment - Disinfection and decommissioning of inactive equipment for surplus - Performance management of equipment (e.g., certification, temperature monitoring of refrigerators and incubators, etc.) | |
| 4. Purchasing & Inventory | - Establishment of an efficient process for purchasing of laboratory equipment, supplies, and reagents - Inventory management of laboratory equipment, supplies, and reagents - Establishment of procedures for receipt of materials and verification of condition upon arrival | |
| 5. Process Management | - Establishment of integrated processes and procedures throughout laboratory path of workflow - Planning and documentation of laboratory processes and workflow - Validation and verification of laboratory processes for efficiency (e.g., to eliminate cross-contamination) and safety - Monitoring of process performance for continuous improvement - Development of quality indicators to monitor laboratory quality management activities, data, and results | |
| 6. Assessments | - Internal laboratory assessments - External laboratory assessments - Regulatory and accreditation inspections/audits (as applicable) - Proficiency Testing (PT) - Alternative Assessment Procedures (AAPs) | |
| 7. Personnel | - Orientation of new laboratory personnel - Training of laboratory personnel - Competency assessments for laboratory personnel - Continuing education and professional development of laboratory personnel - Performance evaluation of laboratory personnel | |
| 8. Customer Satisfaction | - Identification of customers/stakeholders of the laboratory or for the services rendered by the laboratory - Determination of customer/stakeholder expectations of the laboratory’s responsibilities, performance, and deliverables - Measurement of customer/stakeholder satisfaction of the laboratory’s performance and deliverables - Management of customer/stakeholder complaints related to the laboratory’s responsibilities, performance, and deliverables | |
| 9. Occurrence Management | - Identification and reporting of laboratory occurrences - Investigation and documentation of non-conforming events - Implementation of corrective and preventive actions addressing laboratory occurrences - Tracking and analysis of laboratory occurrences and corrective actions - Monitoring of newly implemented corrective actions for effectiveness | |
| 10. Continual Improvement | - Identification and assessment of opportunities for improvement of laboratory operations - Generation of possible solutions for efficient quality management of laboratory operations - Implementation of solutions for effective quality management and efficient laboratory operations - Evaluation of the effectiveness of solutions for quality program and laboratory operations - Integration and sustainment of improvements for quality management and laboratory operations | |
| 11. Documents & Records | - Establishment, maintenance, update, and control of documents related to laboratory activities and operations - Establishment of a Laboratory Document Management System - Maintenance, storage, version control, and retention of records related to laboratory activities - Establishment of a Laboratory Records Management System | |
| 12. Information Management | - Management of access, traceability, retrievability, storage, and flow of laboratory data and information - Management of the confidentiality of information - Management of data security - Management of data integrity - Establishment of procedures for quality control, quality assurance, and reporting of laboratory results | |

# **Recommended QSE Prioritization**

# Table 2 below displays the 12 QSEs of an LQMS adapted from the World Health Organization’s (WHO’s) LQMS Handbook (WHO, 2011), which have been color-formatted to display our recommended prioritization level for each major laboratory type: critical QSEs are shown in red, and beneficial QSEs are shown in yellow.

Table 2: Recommended QSE Prioritization Matrix for LQMS Implementation

| QSE | Testing Laboratories  (e.g., Regulatory Testing) | Product Development & Manufacturing Laboratories | Proficiency Testing Laboratories | Clinical Laboratories | Basic & Applied Research Laboratories |
| --- | --- | --- | --- | --- | --- |
| 1. Organization | **Critical** | **Critical** | **Critical** | **Critical** | **Critical** |
| 2. Facilities & Safety | **Critical** | **Critical** | **Critical** | **Critical** | **Critical** |
| 3. Equipment | **Critical** | **Critical** | **Critical** | **Critical** | **Critical** |
| 4. Purchasing & Inventory | **Critical** | **Critical** | **Critical** | **Critical** | **Beneficial** |
| 5. Process Management | **Critical** | **Critical** | **Critical** | **Critical** | **Beneficial** |
| 6. Assessments | **Critical** | **Critical** | **Critical** | **Critical** | **Beneficial** |
| 7. Personnel | **Critical** | **Critical** | **Critical** | **Critical** | **Critical** |
| 8. Customer Satisfaction | **Critical** | **Critical** | **Critical** | **Critical** | **Beneficial** |
| 9. Occurrence Management | **Critical** | **Critical** | **Critical** | **Critical** | **Beneficial** |
| 10. Continual Improvement | **Critical** | **Critical** | **Critical** | **Critical** | **Beneficial** |
| 11. Documents & Records | **Critical** | **Critical** | **Critical** | **Critical** | **Critical** |
| 12. Information Management | **Critical** | **Critical** | **Critical** | **Critical** | **Beneficial** |

# **Acronyms List**

| AAMC | Association of American Medical Colleges | ISO | International Organization for Standardization |
| --- | --- | --- | --- |
| AAP | Alternative Assessment Procedure | **LQMS** | Laboratory Quality Management System |
| CFR | Code of Federal Regulations | **PT** | Proficiency Testing |
| CLIA | Clinical Laboratory Improvement Amendments | **QA** | Quality Assurance |
| CMS | Centers for Medicare & Medicaid Services | **QMS** | Quality Management System |
| COVID-19 | Coronavirus Disease 2019 | **QSE** | Quality System Essential |
| GLP | Good Laboratory Practices | **WHO** | World Health Organization |

# Supporting References

World Health Organization. (2011). Laboratory quality management system handbook. World Health Organization. Lyon, France. <http://apps.who.int/iris/bitstream/10665/44665/1/9789241548274_eng.pdf>
